# Timing of Expansion of Fragile X Premutation Alleles During Intergenerational Transmission in a Mouse Model of the Fragile X-Related Disorders

**DOI:** 10.3389/fgene.2018.00314

**Published:** 2018-08-10

**Authors:** Xiao-Nan Zhao, Karen Usdin

**Affiliations:** Gene Structure and Disease Section, Laboratory of Cell and Molecular Biology, National Institute of Diabetes and Digestive and Kidney Diseases, National Institutes of Health, Bethesda, MD, United States

**Keywords:** fragile X-related disorders (FXDs), FX-associated tremor and ataxia syndrome (FXTAS), FX-associated primary ovarian insufficiency (FXPOI), fragile X syndrome (FXS), premutation, full mutations, repeat expansion disease, gametogenesis

## Abstract

Fragile X syndrome (FXS) is caused by the maternal expansion of an unstable CGG-repeat tract located in the first exon of the *FMR1* gene. Further changes in repeat number occur during embryogenesis resulting in individuals sometimes being highly mosaic. Here we show in a mouse model that, in males, expansions are already present in primary spermatocytes with no additional expansions occurring in later stages of gametogenesis. We also show that, in females, expansion occurs in the post-natal oocyte. Additional expansions and a high frequency of large contractions are seen in two-cell stage embryos. Expansion in oocytes, which are non-dividing, would be consistent with a mechanism involving aberrant DNA repair or recombination rather than a problem with chromosomal replication. Given the difficulty of replicating large CGG-repeat tracts, we speculate that very large expanded alleles may be prone to contract in the mitotically proliferating spermatagonial stem cells in men. However, expanded alleles may not be under such pressure in the non-dividing oocyte. The high degree of both expansions and contractions seen in early embryos may contribute to the high frequency of somatic mosaicism that is observed in humans. Our data thus suggest an explanation for the fact that FXS is exclusively maternally transmitted and lend support to models for repeat expansion that are based on problems arising during DNA repair.

## Introduction

The fragile X-related disorders (FXDs) result from intergenerational expansions or increases in the number of CGG-repeats in a tandem repeat tract located downstream of the transcription start site of the fragile X mental retardation gene (*FMR1*; MIM ^∗^309550). Premutation (PM) alleles have 55–200 repeats and confer risk of fragile X-associated tremor/ataxia syndrome (FXTAS; MIM #300623) and fragile X-associated primary ovarian insufficiency (FXPOI; MIM #311360). In addition, female PM carriers are at risk of transmitting an *FMR1* allele with >200 repeats to their children. Carriers of such full mutation (FM) alleles have fragile X syndrome (FXS; MIM #300624), the most common heritable cause of intellectual disability and autism. However, not only do male PM carriers not transmit FM alleles to their children, but FXS males who have FM alleles in their somatic cells, only have PM alleles in sperm (Reyniers et al., 1993; Luo et al., 2014; Basuta et al., 2015).

Understanding when and where expansion occurs during intergenerational transfer would help address a number of unresolved questions related to the unusual underlying mutation, including whether the expansion mechanism involves aberrant chromosomal replication or repair and why transmission of FM alleles, and thus FXS, only occurs on maternal transmission. We have generated a mouse model of the FX PM that shows repeat instability reminiscent of what is seen in human PM carriers. This includes having a strong expansion bias and a dependence of these expansions on transcription or the presence of the PM alleles on the active X chromosome (Lokanga et al., 2013). Furthermore, work from other related human Repeat Expansion Disorders suggests that expansions are dependent on some of the same mismatch repair factors that we have shown to be essential for expansion in the PM mouse (Du et al., 2012; Lokanga et al., 2014b; Zhao et al., 2015).

Our previous work demonstrated that different cell types show differences in their propensity to expand (Lokanga et al., 2013). To better understand when expansion occurs during paternal transmission in the FX PM mouse model, we monitored the repeat size in sperm and in different populations of immature male gametes. We also monitored the effect of paternal age on the number of repeats added to alleles transmitted to their offspring. To determine the timing of expansion during maternal transmission, we examined the effect of maternal age on expansion size and monitored the difference between the repeat size in ova and in two-cell embryos after *in vitro* fertilization. Our findings have implications for the mechanism of expansion and may also provide a simple explanation for why large alleles are exclusively maternally transmitted in humans.

## Materials and Methods

### Reagents and Services

All reagents were from Sigma-Aldrich (St. Louis, MO, United States) unless otherwise specified. Collagenase IV was obtained from Thermo Fisher (Waltham, MA, United States), Dispase from STEMCELL Technologies (Vancouver, BC, Canada), Pregnant Mare Serum Gonadotropin (PMSG) from ProSpec-Tany TechnoGene Ltd. (Ness Ziona, Israel), and Cook IVF medium from Cook Medical LLC (Bloomington, IN, United States). Primers were synthesized by Life Technologies (Grand Island, NY, United States). Capillary electrophoresis of fluorescently labeled PCR genotyping products was carried out by the Roy J. Carver Biotechnology Center, University of Illinois (Urbana, IL, United States).

### Mouse Breeding and Maintenance

The generation of the FX PM mice was described previously (Entezam et al., 2007). These mice are on a C57BL/6 background. Mice with a null mutation in Exonuclease 1 (*Exo1*; MIM ^∗^606063), used here as a control for sorting of male gametes, were obtained from Winfried Edelmann (Wei et al., 2003). Mice were maintained in accordance with the guidelines of the NIDDK Animal Care and Use Committee and with the *Guide for the Care and Use of Laboratory Animals* (NIH publication no. 85-23, revised 1996). All data described in this manuscript were obtained using mice for which we had NIH Animal Care and Use Committee (ACUC) approval (ASP# K021-LMCB-18).

### Isolation of Different Testicular Cell Types

Testicular cells were isolated from PM mice and *Exo1* null mice by enzymatic dissociation as previously described (Guan et al., 2009). Briefly, testes were isolated from euthanized 6-month-old male mice with ∼170 repeats. After removal of the tunica albuginea, testes were macerated and treated with 4 mL DMEM/Collagenase/DNase solution, containing 1 mg/mL collagenase IV and 100 U/mL DNase, for 20 min at 37°C in CO_2_ incubator.

The seminiferous tubules were gently pipetted using a truncated 1000 μL tip and collected by centrifugation at 200 *g* for 5 min at room temperature. The tubules were washed three times with PBS. They were then digested with 4 mL Dispase with 100 U/mL DNase for 20 min at 37°C in a CO_2_ incubator with gentle pipetting of the cells every 5 min. The debris was removed by a filtration step using a 70-μm cell strainer and the cells collected by centrifugation at 500 *g* for 5 min at room temperature. The cells were suspended in 3 mL DMEM with 10% FBS and 10% DMSO, transferred to cryovials and frozen using the StrataCooler^®^ Cryo preservation module (San Diego, CA, United States) and stored in liquid nitrogen.

**FIGURE 1:**
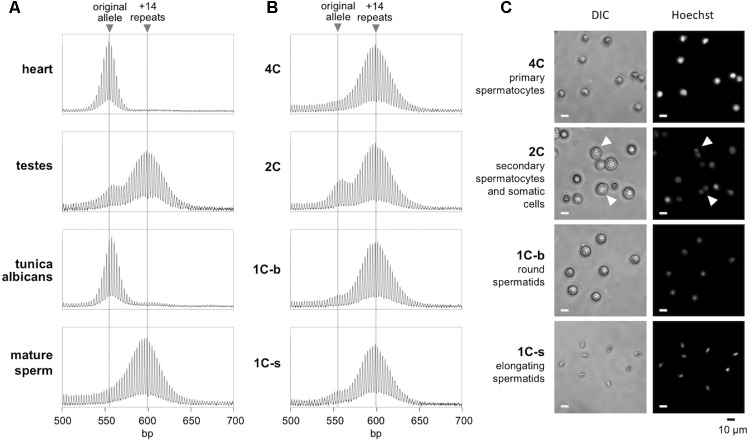
Repeat PCR profiles of different gamete populations in the testes. **(A)** The repeat PCR profile of heart, testes, tunica albicans, and mature sperm from a 6-month-old male mouse. **(B)** The repeat PCR profile of different gamete populations purified by flow cytometry from the same animal as described in the “Materials and Methods” section. **(C)** Morphology of the sorted cell populations. Arrows indicate cells with two distinct nuclei and correspond to secondary spermatocytes just before cytokinesis. Scale bars, 10 μm. 1C-s: small cells with 1C DNA content; 1C-b: big cells with 1C DNA content.

Different testicular cell types were isolated by flow cytometry using a slight modification of a previously published procedure (Hayama et al., 2016). In brief, the frozen cells described above were thawed, suspended in warm DMEM with 10% FBS, and then washed with PBS. Cells were then resuspended in PBS with 5% FBS, counted and diluted to 1 × 10^6^/mL. Cell were stained with 5 mg/mL Hoechst 33342 in PBS with 5% FBS at 37°C for 45 min. Cells were then washed with PBS and resuspended in PBS with 5% FBS. A FACS Aria II (BD Biosciences, San Jose, CA, United States) flow sorter was used for sorting. Hoechst fluorescence was detected with a 450-nm band-pass filter for blue fluorescence or a 675-nm band-pass filter for red fluorescence (Sherman et al., 1985). Sorted cells were collected in PBS with 5% FBS. To identify haploid populations, we compared normal mice with *Exo1^-/-^* adult males. These mice cannot complete meiosis and thus lack haploid sperm (Wei et al., 2003). Sorting for blue versus red (450-nm versus 675-nm band-pass emission, respectively) and for size, revealed four subpopulations in normal mice. Subpopulation 1 contains prophase spermatocytes with a 4C DNA content. Subpopulation 2 included secondary spermatocytes and some somatic cells with a 2C content. Subpopulation 3 included post-meiotic haploid round spermatids with a 1C DNA content; and subpopulation 4 included elongated spermatids and spermatozoa with a 1C DNA content. We examined the purity of each subpopulation using fluorescence microscopy (**Figure 1C**). The purity of subpopulations 1, 3, and 4 were estimated to be over 90%. The morphology of the cells in each subpopulation matched a previous report for mouse testicular cells (Bastos et al., 2005). Subpopulations 3 and 4 were lacking in the *Exo1^-/-^* mice, consistent with these populations comprising haploid gametes absent in *Exo1^-/-^* mice (**Supplementary Figure S1**).

### Collection of Oocytes and Embryos

Ova were collect from superovulated 6-week-old and 10-week-old female FX PM mice using standard procedures (Nagy et al., 2003). Briefly, the mice were injected first with 5 IU PMSG and 47 h later injected with 5 IU human chorionic gonadotropin (hCG). Twenty hours later the females were euthanized and the oviducts transferred to a clean dish. The ampullae were removed and placed in a dish with Cook IVF medium. Fine forceps and needles were used to release the cumulus oocyte masses. Half of the oocytes were collected using an oocyte handling pipette and placed in individual aliquots of 20 μL PBS and frozen at -80°C. The remaining oocytes were transferred to a fertilization dish with Cook medium and incubated with sperm from a wild-type male for 4 h at 37°C. After fertilization, embryos were washed and incubated in Cook medium at 37°C overnight. The 2-cell embryos were collected in individual aliquots of 20 μL PBS and frozen at -80°C.

### DNA Isolation

Genomic DNA from mouse tails was extracted using KAPA Mouse Genotyping Kit (KAPA Biosystems, Wilmington, MA, United States). Genomic DNA from other tissues was extracted using a Maxwell^®^16 Mouse tail DNA purification kit (Promega, Madison, WI, United States) according to the manufacturer’s instructions. DNA from oocytes and embryos were prepared as follows. Oocytes and embryos in 20 μL PBS were incubated at 37°C for 10 min and then placed on dry ice for 10 min. This thawing and freezing cycle was repeated three times and followed by digestion with 2 mg/mL Proteinase K at 55°C for 2 h. After Proteinase K treatment, the sample was incubated at 95°C for 10 min. Sperm was isolated from mouse epididymis using standard procedures. DNA from sperm and from different testicular cell types was isolated as follows. Sperm and testicular cells were collect by centrifuge at 500 *g* for 5 min and fully mixed with 300 μL ATL buffer (Qiagen, Hilden, Germany) with 0.55 mg/mL Proteinase K and 30 mM DTT by vortexing for 10 s. After incubation at 55°C overnight, 90 μL of 5 M NaCl was added into the mixture, followed by centrifugation at 13,000 *g* for 10 min. The supernatant was transferred to a new tube and mixed with 390 μL ethanol. After incubation at -20°C for 1 h, DNA was pellet by centrifugation at 13,000 *g* for 10 min. After washing with 70% ethanol, the DNA pellet was dissolved in TE at 55°C for 15 min.

### Genotyping and Analysis of Repeat Number

Exonuclease 1 genotyping was carried out with KAPA mouse genotyping kit (KAPA Biosystems, Wilmington, MA, United States) according to the manufacturer’s instructions with either the Exo1A (5′-CTCTTGTCTGGGCTGATATGC-3′)/Exo1B (5′-ATGGCGTGCGTGATGTTGATA-3′) primer pair to detect the WT allele and Exo1C (5′-AGGAGTAGAAGTGGCGCGAAGG-3′)/Exo1B to detect the mutant allele (Winfried Edelmann’s lab). PM allele genotyping was carried out using a fluorescent PCR assay with FAM-labeled FraxM4 (FAM-5′-CTTGAGGCCCAGCCGCCGTCGGCC-3′) and FraxM5 (5′-CGGGGGGCGTGCGGTAACGGCCCAA-3′) primer pair as described previously (Zhao et al., 2016). Briefly, 0.5 μM each of the primers FraxM4 and FraxM5 were added to reaction mixes containing 10 ng DNA, 1× KAPA2G Fast HotStart Genotyping Mix (KAPA Biosystems, Wilmington, MA, United States), 2.5 M Betaine and 2% DMSO. PCR parameters were 1× 95°C for 10 min, 35× (95°C for 30 s, 65°C for 30 s, and 72°C for 70 s), followed by 1× 72°C for 10 min. Nested PCR for oocytes and embryos samples was carried out using Expand^TM^ High Fidelity PCR System (Roche) as previously described (Lokanga et al., 2015). The first round of PCR was carried out using the Frax-C (5′-GCTCAGCTCCGTTTCGGTTTCACTTCCGGT-3′) and Frax-F (5′-AGCCCCGCACTTCCACCACCAGCTCCTCCA-3′) primer pair (Fu et al., 1991) in a 20 μL reaction. The PCR mix containing 1× Hi-Fi buffer with 2.5 M Betaine, 2% DMSO, 0.2 mM dNTPs, 0.5 μM of each primer and 0.07 U/μL of the DNA polymerase mix. PCR parameters were 1 × 95°C for 10 min, 35× (95°C for 1 min, 65°C for 1 min, and 72°C for 5 min), followed by 1× 72°C for 10 min. One microliter of this PCR reaction was then subjected to a second round of PCR using FAM-Frax-M4 and Frax-M5. The PCR products were resolved by capillary electrophoresis on an ABI Genetic Analyzer (Lokanga et al., 2013). The resultant fsa file was then displayed using a custom R script (Hayward et al., 2016) that is available on request. Fisher’s exact test and Student’s *t*-test were carried out using the GraphPad QuickCalcs website^1^. Mann–Whitney *U* tests were carried out using the Vassarstats website ^2^.

## Results

### Male Germline Expansions Are Confined to the Early Stages of Gametogenesis

To determine the timing of expansion during male gametogenesis, we isolated testicular cells from 6-month-old males with ∼170 repeats and sorted the germ cell population according to their DNA content and size as described in the Section “Materials and Methods.” We prepared genomic DNA from each gamete subpopulation along with the tunica albicans, mature sperm and the remaining intact testicle from each animal. In parallel, we also isolated DNA from heart, an organ we have previously shown to have no post-natal somatic expansions (Lokanga et al., 2013). We then subjected the samples to PCR using primers flanking the repeat and analyzed the repeat PCR profiles by high-resolution capillary electrophoresis. Since the heart does not undergo expansion, a single peak is seen in the repeat PCR profile that corresponds to the original inherited allele (**Figure 1**). In contrast, the repeat PCR profile of a typical testis sample shows two peaks, one that is similar in size to the allele in heart and a second peak that has a size consistent with it having ∼14 repeats more than the allele in heart. The tunica albicans shows a repeat PCR profile similar to that in heart, indicating little or no expansion in these somatic cells. However, the mature sperm showed a single peak similar to the +14 repeat peak seen in the testis. This is consistent with all of the mature gametes having alleles that have expanded. To ascertain when this expansion had occurred, we examined the various germ cell subpopulations. The 4C and 1C subpopulations corresponding to primary spermatocytes and haploid spermatids, respectively, each had a single peak corresponding to the larger peak seen in mature sperm (**Figure 1B**). Perhaps unsurprisingly, the repeat PCR profile for the 2C population, a population that contains a mixture of somatic cells and secondary spermatocytes, showed a mixture of both alleles. Since no unexpanded alleles could be detected in the primary spermatocytes and the allele size did not increase in more mature gametes, our data suggest that expansion occurs prior to meiosis in the dividing spermatogonia.

**FIGURE 2:**
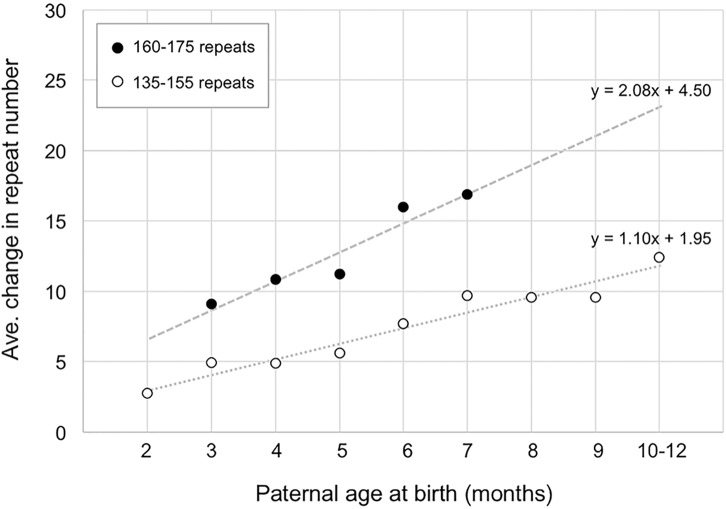
The relationship between paternal age and the number of repeats added to the progeny allele. The average number of repeats added to the progeny alleles was plotted as a function of paternal age (open circles, fathers with 135–155 repeats; filled circles, fathers with 160–175 repeats). The data for fathers with 135–155 repeats represents a total of 372 PM pups from 24 fathers. Each time point comprises an average of at least 17 animals. The data for fathers with 160–175 repeats represents a total of 58 PM pups from 7 fathers with each time point representing an average of at least 10 animals.

The fact that ∼14 repeats have been added to the PM allele in the gametes of 6-month-old males does not mean that all 14 repeats were added simultaneously. As can be seen in **Figure 2**, when the number of repeats added to the offspring alleles is plotted as a function of paternal age, it is apparent that for males with 160–175 repeats, the average size of the alleles that they transmit increases by ∼2.1 repeats/month (**Figure 2**). In the case of paternal alleles with 135–155 repeats, an average of ∼1.1 repeats are added to the transmitted allele each month. However, while most expansions are small, involving as few as 1 or 2 repeats, larger expansions are also sometimes seen (**Supplementary Figure S2**).

### Expansion Occur in Oocyte During Arrest in Meiotic Prophase I

**FIGURE 3:**
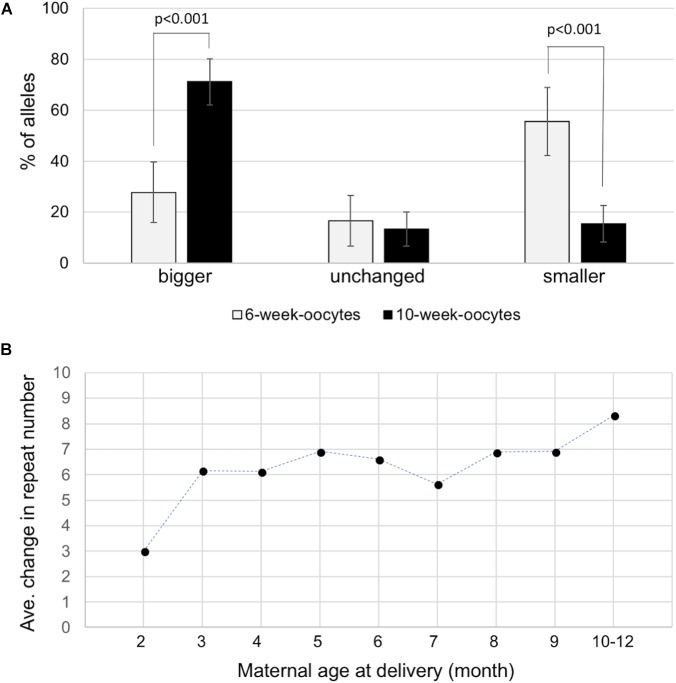
Expansion in the female germline. **(A)** Distribution of allele classes in ova retrieved from 6-week-old and 10-week-old females with 155–165 repeats. Alleles that were bigger than, the same as or smaller than the maternal alleles are plotted. **(B)** The average number of repeats added to the progeny alleles was plotted as a function of maternal age at the birth of each litter. The data represent a total of 231 KI pups in 79 litters from 14 mothers with 145–155 repeats. Each time point represents the average of at least 18 animals.

We next sought to determine whether expansions occurred during the proliferative phase involved in the establishment of the primordial follicle pool or post-natally in the prophase I-arrested oocyte. To do this we isolated ova from 6-week-old and 10-week-old females with 155–165 repeats and compared the repeat size in individual ova using nested PCR as described in the Section “Materials and Methods.” We analyzed 54 ova from 6-week-old animals and 107 ova from 10-week-old animals. As can be seen in **Figure 3A**, the number of ova with alleles larger than the original maternal allele was significantly higher in 10-week-old females than in 6-week-old females. Analysis of the number of repeats added to the alleles of the offspring of 2- and 3-month-old mothers was also consistent with the increase seen in ova with 2-month-old mothers having pups that had gained an average of three repeats compared to an average of 6.2 repeats in the pups of 3 month-old mothers (*p* = 0.0002; **Figure 3B** and **Supplementary Figure S3**). The average number of repeats added to offspring alleles continued to increase with increasing maternal age with the average allele size being significantly larger in 10-month-old mothers than in 3-month-old ones (*p* = 0.026). However, the rate at which alleles increase in size is slower than in the progeny of younger mothers. The transition to a slower expansion rate roughly coincides with the shift from the use of a wave of activated primordial follicles that contributes to fertility from puberty into early adulthood, to the second wave of follicles that serves as the sole source of oocytes for fertilization in later reproductive life in mice and humans (Zheng et al., 2014). The follicles in the first wave originate in the ovarian medulla while the second wave of follicles originates in the cortex, with location in the ovary suggested to be the determinant of the timing of activation rather than any difference in the timing of meiotic arrest (Cordeiro et al., 2015). Follicles in the medulla and cortex differ in a number of ways apart from their location in the ovary (Zheng et al., 2014; Hummitzsch et al., 2015) and it may be that the two populations of oocytes are exposed to different signals that affect expansion rate. In addition, contractions occur throughout the reproductive life of these animals (Zhao and Usdin, 2014, 2015) that can result in a broader distribution of alleles that obscures any expansions that do occur.

### Expansion and Contraction Also Occurs in the Very Early Embryo

**FIGURE 4:**
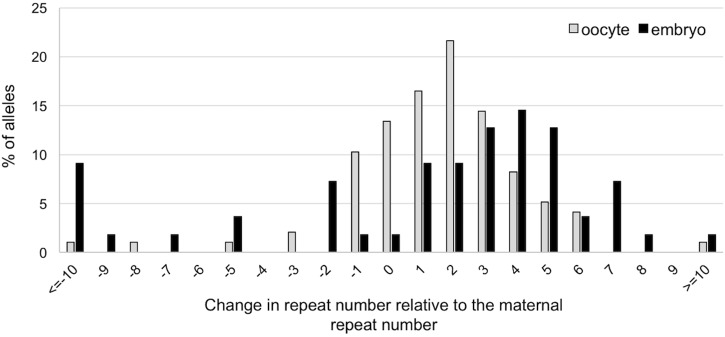
Difference in repeat number changes in ova and 2-cell embryos. The size of the alleles in each ovum and embryo was determined by nested PCR. Alleles were then analyzed in terms of the change in repeat number relative to the maternal allele. The distribution of the changes in repeat number of expanded allele in embryos is significantly different from the distribution on oocytes (Mann–Whitney; *p* = 0.001; *t*-test; *p* = 0.01), with embryos showing evidence of additional repeats added to the transmitted allele. The difference between ova and embryos with respect to the number of large contractions was also significant (Fisher’s exact test; *p* = 0.026).

To evaluate whether expansion also occurs in the early embryo we compared the size of the alleles obtained in 107 ova obtained from 10-week-old females and in 55 two-cell embryos produced from ova retrieved at the same time from the same females. A similar high proportion of expansions was seen in the ova and the embryos. However, as can be seen in **Figure 4**, the embryos show a rightward shift in the distribution of the number of repeats added to the progeny alleles relative to the oocytes. This would be consistent with embryos having been subject to additional expansion events (*U*-test, *p* = 0.001; *t*-test, *p* = 0.01). Thus, additional expansions can occur in the ∼20 h between the time the ova were harvested and the 2-cell embryo stage. The number of large contractions (>10 repeats) was also significantly higher in the 2-cell embryos than in the ova (*p* = 0.026).

## Discussion

In this study we examined the timing of expansion during intergenerational transmission of FX PM alleles in a mouse model. We showed that expansions occur during male and female gametogenesis as well as in the early embryo. We demonstrated that expansions are already present in primary spermatocytes of FX PM mice with no additional expansions being seen in more mature gametes (**Figure 1**). The repeat PCR profile of the expanded allele in gametes is consistent with the mathematical modeling of the continuous small but high frequency expansions seen in a mouse model of HD (Mollersen et al., 2010). This would be consistent with our demonstration that increasing paternal age is associated with the addition of an average of ∼1.1 repeats with each expansion event in fathers with 130–155 repeats (**Figure 2**). However, these events occur relatively frequently, slightly more than once a month for these fathers. Fathers with 160–175 repeats show an addition of an average of ∼2 repeats per month (**Figure 2**). Whether this represents expansions that also occur once a month but are larger, or single repeat expansions that are now more frequent, is unclear. Small, but frequent expansions are also seen in mouse models (Mollersen et al., 2010) and human cell models for other Repeat Expansion Diseases. Furthermore, a similar threshold for an increase in the rate of repeat accumulation with increasing repeat number has also been seen in some of these model systems (Ditch et al., 2009; Ku et al., 2010; Du et al., 2012, 2013). The basis of this threshold is unknown. It may be that it reflects the thermodynamic threshold for the stable formation of an intermediate in the expansion process or the cooperative nature of binding of some of the proteins thought to be important for expansion.

Expansion that is limited to the early stages of spermatogenesis differs from what has been reported for HD, where expansions are seen in the haploid gamete in a mouse model (Kovtun and McMurray, 2001) and in both spermatogonia and haploid gametes in humans with HD (Yoon et al., 2003). The timing of expansion in our FXD mouse model more closely resembles the timing of expansion reported for a mouse model of Myotonic Dystrophy Type 1, a CTG-repeat expansion disease, where expansion was found to be confined to the spermatogonia (Savouret et al., 2004).

In contrast to expansion in dividing cells in the male germ line, our data suggests that expansion during oogenesis occurs in non-dividing oocytes, since the ova isolated from older mothers have more expansions than the ova from younger mothers (**Figure 3A**). Expansion in the oocyte is also reflected in the fact that the number of repeats added to expanded alleles in the progeny of older mothers is higher than the repeat number added in the progeny of younger mothers (**Figure 3B**). Since female germ cells enter meiosis in the prenatal embryo and do not replicate their genomes again until after fertilization (Borum, 1961), the increased expansion frequency in older mothers would be consistent with the idea that the expansions in oocytes result from problems arising during DNA repair/recombination rather than chromosomal replication *per se*. These results are consistent with reports of a maternal age effect for expansion risk in human females (Yrigollen et al., 2014). A replication-independent mechanism for expansion is also consistent with the fact that expansion in post-mitotic neurons is seen in other repeat expansion diseases and mouse models of these diseases (Watanabe et al., 2000; Hashida et al., 2001; Kennedy et al., 2003; Wheeler et al., 2003; Shelbourne et al., 2007; Gonitel et al., 2008; Kovalenko et al., 2012).

It is possible that expansion proceeds via a replication-based mechanism in dividing cells, like spermatogonia and/or the early embryo, as some have suggested (Gerhardt et al., 2014a,b), and via a repair/recombination-based mechanism in non-dividing cells. However, in the FX PM mouse model, expansions in all cell types examined have the same requirement for mismatch repair proteins and base excision repair proteins (Lokanga et al., 2014b, 2015; Zhao et al., 2015, 2016), and all require the PM allele to be transcribed (Lokanga et al., 2014a). Thus, if different mechanisms operate in different cell types in the mouse model, they must all share these somewhat unusual requirements.

While most of the expansions we see in mice are small, women with PM alleles frequently transmit very much bigger alleles to their children, particularly when the maternal allele has ∼90–100 repeats (Nolin et al., 2015). However, since human oocytes would have decades to accumulate expansions and our data predicts that the rate at which additional repeats are added would increase exponentially with time, it is possible that FM alleles result from small but frequent expansions. A simple modeling of this process is shown in **Supplementary Figure S4**. In addition, in a small fraction of both paternal and maternal transmissions, the transmitted allele is much larger than average (>2 standard deviations away from the mean; **Supplementary Figures S2, S3**). These events likely involve the same basic mechanism as the smaller expansions since they are eliminated by mutations in genes that also eliminate the smaller expansions (Lokanga et al., 2014b; Zhao et al., 2015, 2016). It is thus possible that these larger expansions also contribute to the growth of the transmitted allele with maternal age.

In humans, PM alleles with 55–59 repeats are ∼4 times more likely to expand on paternal transmission than on maternal transmission (Sullivan et al., 2002). Male mice also show more expansions than females (Lokanga et al., 2013, 2014a; Zhao and Usdin, 2014). However, the fact that expansion during spermatogenesis occurs in cells that undergo many rounds of cell division may explain why human PM males do not transmit FM alleles to their children and men with FM alleles in their somatic cells only have PM alleles in their sperm (Reyniers et al., 1993; Luo et al., 2014; Basuta et al., 2015). CGG-repeats are notoriously difficult to replicate (Usdin and Woodford, 1995; Samadashwily et al., 1997; Voineagu et al., 2009; Yudkin et al., 2014), likely because they form stable secondary structures that block DNA synthesis (Usdin and Woodford, 1995; Voineagu et al., 2009). Thus, large expanded alleles in spermatogonia may be under continuous pressure to contract, perhaps by strand-slippage during replication. While selection against large alleles in sperm is also theoretically possible, FM males are not sterile (Reyniers et al., 1993; Luo et al., 2014; Basuta et al., 2015). This would favor the idea of early contraction.

Expansions in the low FM range (∼200–300 repeats) are paternally transmitted in mice (Entezam et al., 2007). The fact that male mice transmit FM alleles while men do not, may be related to the smaller number of spermatogonial stem cell (SSC) divisions in adult mice relative to humans. For example, the sperm of 9-month-old male mice have undergone ∼26 stem cell divisions compared to ∼360 for human males at 30 years of age (Drost and Lee, 1995). The absence of replication pressure in oocytes may mean that longer repeats are more easily tolerated and thus less likely to contract, thus accounting for why FM alleles can be maternally, but not paternally, transmitted in humans.

In addition to expansion during gametogenesis, we also show that average increase in repeat number is larger in the 2-cell stage embryos than it is in the ova (**Figure 4**), suggesting that expansions can also occur in the early embryo. Interestingly, base excision repair, a process we have shown to be crucial to expansion (Lokanga et al., 2015), is stimulated on fertilization (Lord and Aitken, 2015). We have also previously shown that expansion is dependent on MutSβ and MutSα (Lokanga et al., 2014b; Zhao et al., 2015, 2016), proteins that are highly expressed in pluripotent stem cells (Ku et al., 2010; Momcilovic et al., 2010; Osman et al., 2010; Seriola et al., 2011; Tichy et al., 2011; Du et al., 2012; Aranda et al., 2014). The high level of expression of these proteins may contribute to the propensity of PM alleles to expand in the early embryo. However, levels of these mismatch repair proteins are thought to drop precipitously on differentiation (Ku et al., 2010; Momcilovic et al., 2010; Osman et al., 2010; Seriola et al., 2011; Tichy et al., 2011; Du et al., 2012; Aranda et al., 2014) and this may limit expansions in differentiated cells as reported for other Repeat Expansion Diseases (Ku et al., 2010; Du et al., 2012, 2013). Since we have been unable to detect clear evidence of expansion in human embryonic stem cells with large unmethylated alleles (Zhou et al., 2016), it may be that expansion is confined to very early embryogenesis.

In addition to larger expansions seen in the 2-cell embryo, there is also a significant increase in the number of large contractions relative to the oocytes (**Figure 4**). It might be that the requirement for rapid cell division coupled with the difficulty in replicating large CGG repeats (Usdin and Woodford, 1995; Voineagu et al., 2009; Yudkin et al., 2014), means that large alleles in early embryos, like those in spermatogonia, are under pressure to contract. These contractions may contribute to the repeat size mosaicism that is sometimes seen in mice and, perhaps by extension, in humans as well (Nolin et al., 1994).

## Ethics Statement

All data described in this manuscript were obtained using mice for which we had NIH Animal Care and Use Committee (ACUC) approval (ASP# K021-LMCB-18).

## Author Contributions

KU and X-NZ designed the experiments, analyzed the data, and wrote the manuscript. X-NZ performed the experiments.

## Conflict of Interest Statement

The authors declare that the research was conducted in the absence of any commercial or financial relationships that could be construed as a potential conflict of interest. The reviewer RFB and handling Editor declared their shared affiliation.

## Footnotes

1 http://www.graphpad.com/quickcalcs

2 http://vassarstats.net

## Supplementary Material

FIGURE S1 Flow-cytometry analysis of Hoechst 33342-stained cells. Flow-cytometry analysis of testicular cells isolated from 6-month-old Exo1^-/-^ **(A)** and FX PM **(B)** males. The left side of panels **(A,B)** shows the Forward Scatter (FSC)/Side Scatter (SSC) profiles. The FSC parameter reflects cell size, while the SSC parameter reflects the cellular complexity. The right side of panels **(A,B)** show the Blue versus Red profiles with the gates used for collection. **(C)** The FSC area (FSC-A) plot of the cell size distribution of the 1C cell population in the FX PM male showing the proportion of small cells with 1C DNA content (1C-s) and big cells with 1C DNA content (1C-b).

FIGURE S2 Box plots showing the effect of paternal age on repeat length changes in the progeny (refers to **Figure 2**). The plots were generated using the default settings of the geom_boxplot function of the R library ggplot2 showing the median, a box containing the 25th to 75th quantile data points, and whiskers extending to data points within 1.5× Interquartile Range. Data points outside this range are shown individually.

FIGURE S3 Box plots showing the effect of maternal age on repeat length changes in the progeny (refers to **Figure 3B**). The plots were generated as described in the legend to **Supplementary Figure S2**. The progeny allele distribution of 2 and 3-month-old mothers were statistically different by *t*-test (*p* = 0.0002) and for 3-month-old and 10-month-old mothers (*p* = 0.026).

FIGURE S4 Simple model for the generation of alleles with large repeat numbers by the high frequency of small expansions. The change in the number of repeats with time is plotted for a starting allele with 100 repeats using the assumption that the average repeat number added to this allele is either initially 1 repeat/month, increasing by 1 repeat/month for every 50 repeats added to the original allele or 1 repeat every 2 months, increasing by 1 repeat every 2 months for every 50 repeats added. This simplistic scenario does not include corrections for contractions and is just meant to illustrate that very large alleles can potentially arise in oocytes via small but frequent expansions over the years between birth and adulthood in human females. Thus, even small expansions that occur less frequently than once a month, could readily generate FM alleles in the interval between birth and conception.
