# Supplementary figures and images for: Timing of Expansion of Fragile X Premutation Alleles During Intergenerational Transmission in a Mouse Model of the Fragile X-Related Disorders
Source: Front Genet. 2018 Aug 10;9:314. doi: 10.3389/fgene.2018.00314 (PMC6096447; doi:10.3389/fgene.2018.00314)

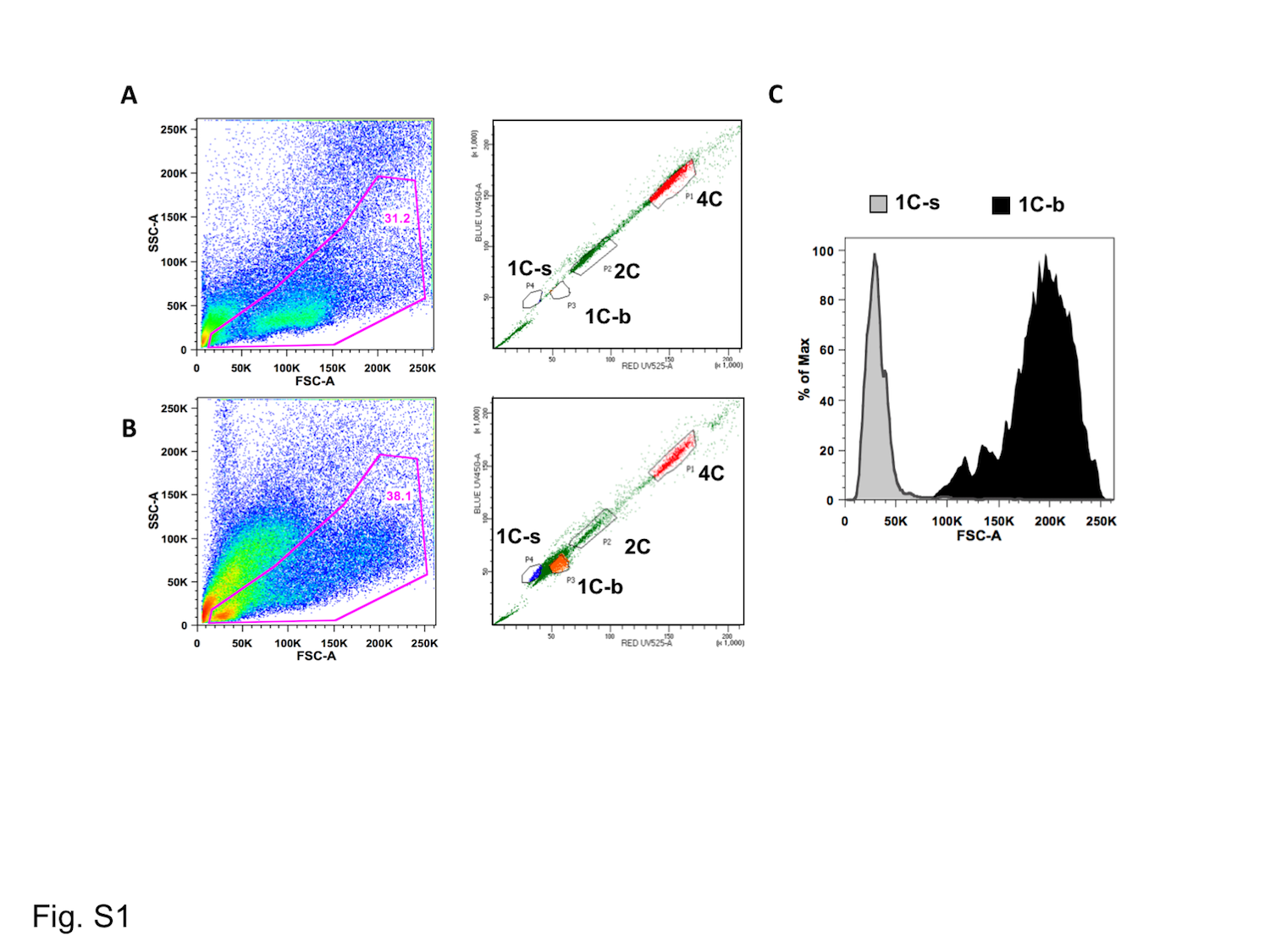

Supplement: FIGURE S1 — Flow-cytometry analysis of Hoechst 33342-stained cells. Flow-cytometry analysis of testicular cells isolated from 6-month-old Exo1-/- (A) and FX PM (B) males. The left side of panels (A,B) shows the Forward Scatter (FSC)/Side Scatter (SSC) profiles. The FSC parameter reflects cell size, while the SSC parameter reflects the cellular complexity. The right side of panels (A,B) show the Blue versus Red profiles with the gates used for collection. (C) The FSC area (FSC-A) plot of the cell size distribution of the 1C cell population in the FX PM male showing the proportion of small cells with 1C DNA content (1C-s) and big cells with 1C DNA content (1C-b). [file Image_1.TIFF]

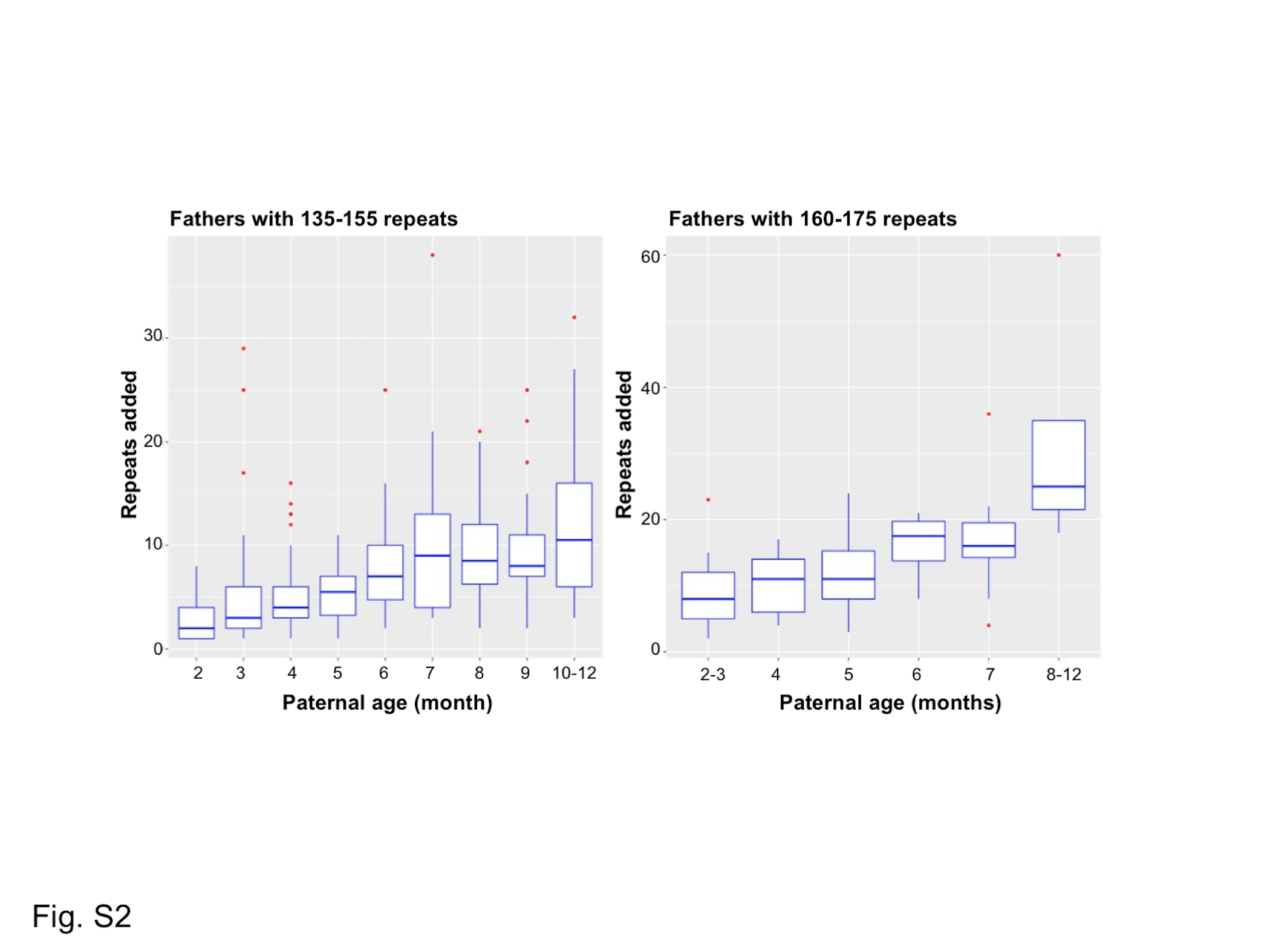

Supplement: FIGURE S2 — Box plots showing the effect of paternal age on repeat length changes in the progeny (refers to Figure 2). The plots were generated using the default settings of the geom_boxplot function of the R library ggplot2 showing the median, a box containing the 25th to 75th quantile data points, and whiskers extending to data points within 1.5× Interquartile Range. Data points outside this range are shown individually. [file Image_2.TIFF]

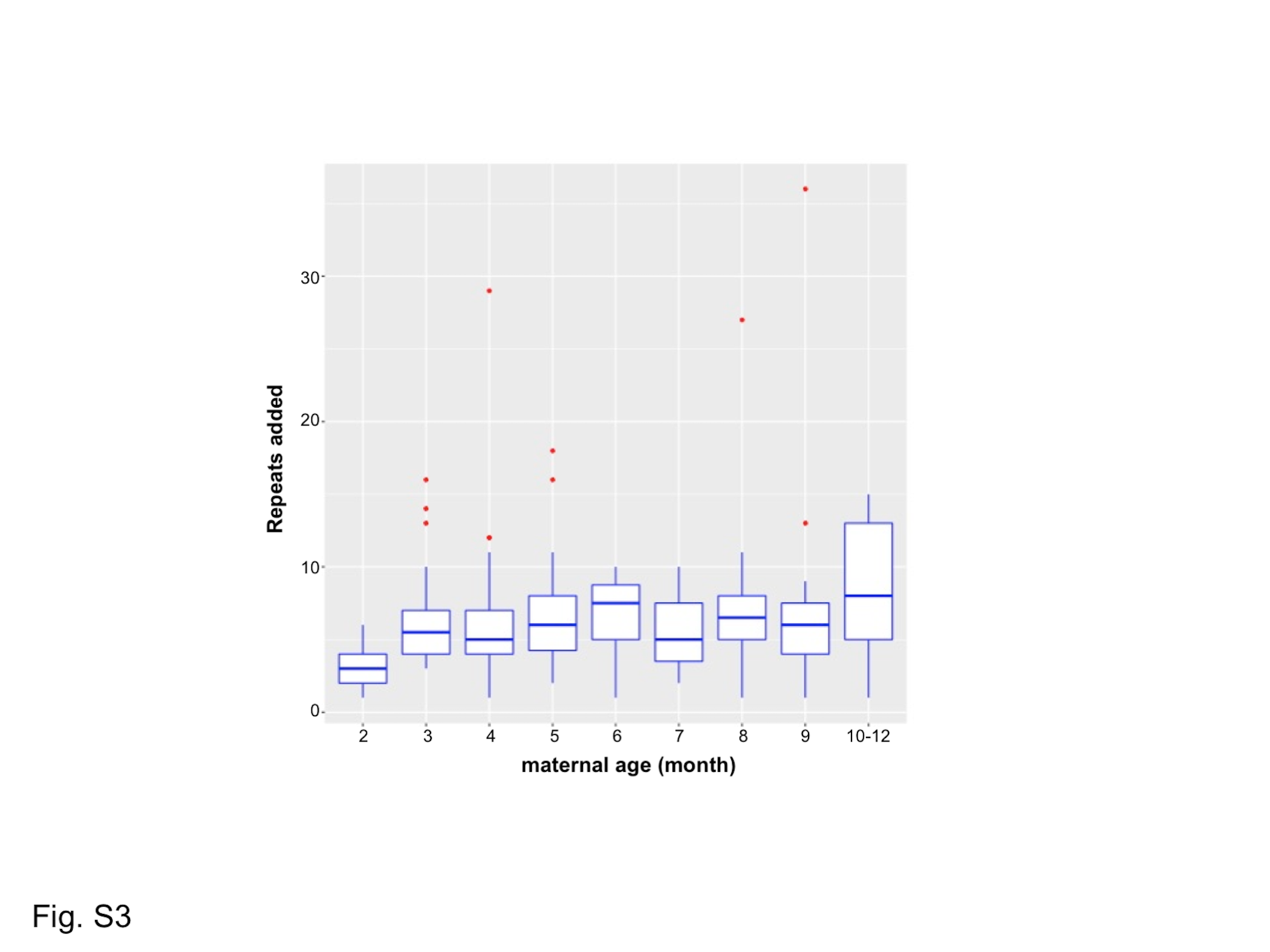

Supplement: FIGURE S3 — Box plots showing the effect of maternal age on repeat length changes in the progeny (refers to Figure 3B). The plots were generated as described in the legend to Supplementary Figure S2. The progeny allele distribution of 2 and 3-month-old mothers were statistically different by t-test (p = 0.0002) and for 3-month-old and 10-month-old mothers (p = 0.026). [file Image_3.TIFF]

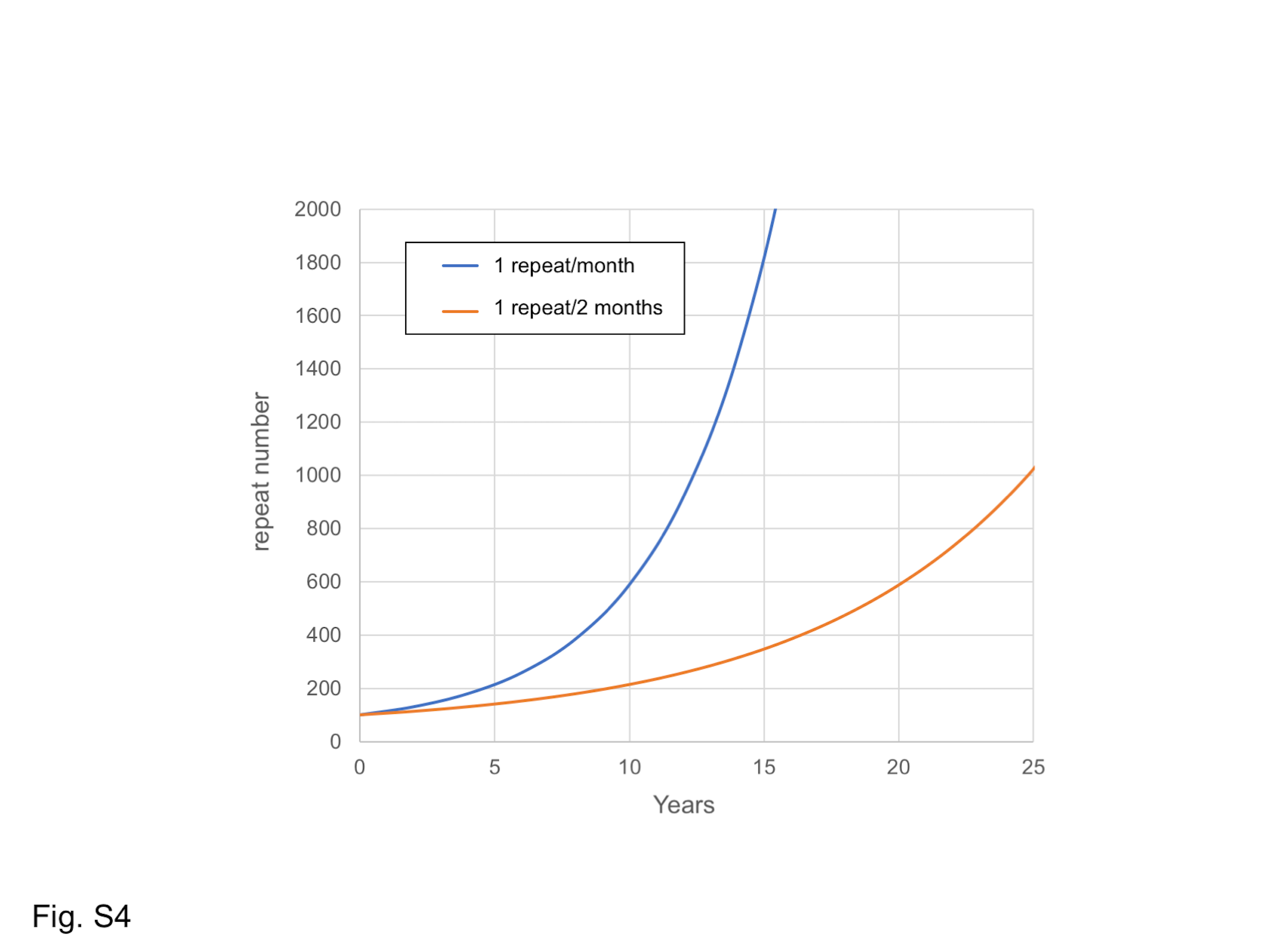

Supplement: FIGURE S4 — Simple model for the generation of alleles with large repeat numbers by the high frequency of small expansions. The change in the number of repeats with time is plotted for a starting allele with 100 repeats using the assumption that the average repeat number added to this allele is either initially 1 repeat/month, increasing by 1 repeat/month for every 50 repeats added to the original allele or 1 repeat every 2 months, increasing by 1 repeat every 2 months for every 50 repeats added. This simplistic scenario does not include corrections for contractions and is just meant to illustrate that very large alleles can potentially arise in oocytes via small but frequent expansions over the years between birth and adulthood in human females. Thus, even small expansions that occur less frequently than once a month, could readily generate FM alleles in the interval between birth and conception. [file Image_4.TIFF]
